# Comparison of the killing effects between nitrogen-doped and pure TiO_2_ on HeLa cells with visible light irradiation

**DOI:** 10.1186/1556-276X-8-96

**Published:** 2013-02-22

**Authors:** Zheng Li, Xiaobo Pan, Tianlong Wang, Pei-Nan Wang, Ji-Yao Chen, Lan Mi

**Affiliations:** 1 Key Laboratory of Micro and Nano Photonic Structures (Ministry of Education), Department of Optical Science and Engineering, Shanghai Ultra-Precision Optical Manufacturing Engineering Center, Fudan University, 220 Handan Road, 200433, Shanghai, China; 2 State Key Laboratory of Surface Physics, Department of Physics, Fudan University, 220 Handan Road, 200433, Shanghai, China

**Keywords:** Nitrogen-doped TiO_2_, Visible-light-activated, Photodynamic therapy, Reactive oxygen species

## Abstract

The killing effect of nitrogen-doped titanium dioxide (N-TiO_2_) nanoparticles on human cervical carcinoma (HeLa) cells by visible light photodynamic therapy (PDT) was higher than that of TiO_2_ nanoparticles. To study the mechanism of the killing effect, the reactive oxygen species produced by the visible-light-activated N-TiO_2_ and pure-TiO_2_ were evaluated and compared. The changes of the cellular parameters, such as the mitochondrial membrane potential (MMP), intracellular Ca^2+^, and nitrogen monoxide (NO) concentrations after PDT were measured and compared for N-TiO_2_- and TiO_2_-treated HeLa cells. The N-TiO_2_ resulted in more loss of MMP and higher increase of Ca^2+^ and NO in HeLa cells than pure TiO_2_. The cell morphology changes with time were also examined by a confocal microscope. The cells incubated with N-TiO_2_ exhibited serious distortion and membrane breakage at 60 min after the PDT.

## Background

In recent years, semiconductor titanium dioxide (TiO_2_) was noticed as a potential photosensitizer in the field of photodynamic therapy (PDT) due to its low toxicity, high stability, excellent biocompatibility, and photoreactivity [1-4]. The electrons in the valence band of TiO_2_ can be excited to the conduction band by ultraviolet (UV) radiation with the wavelength shorter than 387 nm (corresponding to 3.2 eV as the band gap energy of anatase TiO_2_), thus resulting in the photoinduced hole-electron pairs. These photoinduced electrons and holes can interact with surrounding H_2_O or O_2_ molecules and generate various reactive oxygen species (ROS, such as superoxide anion radical O_2_^ ·−^[5], hydroxyl radical OH · [6], singlet oxygen ^1^O_2_[7], and hydrogen peroxide H_2_O_2_[8]), which can react with biological molecules, such as lipids, proteins, and DNA, cause their damages, and eventually kill cancer cells [1,9,10].

However, the pure TiO_2_ can only be excited by UV light which is harmful and hinders its practical applications [11]. Fortunately, recent studies have reported that the optical absorption of TiO_2_ in the visible region could be improved by doping [12-14] or dye-adsorbed methods [15,16], which will facilitate the application of TiO_2_ as a photosensitizer for PDT. In our previous study [10], we enhanced the visible light absorption of TiO_2_ by nitrogen doping and found that the nitrogen-doped TiO_2_ (N-TiO_2_) showed much higher visible-light-induced photokilling effects on cancer cells than the pure TiO_2_.

Although great efforts have been made to prepare doped TiO_2_ with visible light absorption, the underlying mechanism of the killing effects of photoactivated TiO_2_ on cancer cells has not yet been investigated in details. It is unclear how the TiO_2_ interacts with the cancer cells, and what are the differences for their photokilling effects between pure and doped TiO_2_. For possible medical applications of N-TiO_2_, it is of crucial importance to understand the killing effect of N-TiO_2_ on cancer cells and the mechanism of cell damages induced by PDT.

As ROS has been claimed to be of major importance for various kinds of PDT [17-20], the time-dependent ROS productions during visible light irradiation were evaluated in this work for both N-TiO_2_ and TiO_2_ in aqueous suspensions. The productions of different ROS species, such as O_2_^ ·−^, H_2_O_2_, and OH·, were also studied. Furthermore, a systematic comparison of the intracellular parameters with N-TiO_2_ and TiO_2_ nanoparticles as photosensitizers for PDT was investigated. The changes of mitochondrial membrane potential (MMP), intracellular Ca^2+^, and nitrogen monoxide (NO) concentrations with time after the PDT were measured. The relationships between these parameters were discussed. The morphological changes of cytoskeletons after irradiation were also examined by a confocal microscope at different times after the PDT. The killing effects between pure and nitrogen-doped TiO_2_ were compared.

## Methods

### Preparation and characterization of N-TiO_2_ samples

The details of preparation of N-TiO_2_ nanoparticles were described in our previous paper [10]. Briefly, The anatase TiO_2_ nanoparticles (particle size <25 nm; Sigma-Aldrich, St. Louis, MO, USA) were calcined at a flow rate of 3.5 L/min in ammonia atmosphere at 550°C for 20 min to produce the N-TiO_2_ nanoparticles. The crystalline phases of the N-TiO_2_ nanoparticles were determined by Raman spectra to be anatase. The ultraviolet-visible (UV/Vis) diffuse reflectance absorption spectra (Additional file 1: Figure S1) of the N-TiO_2_ and TiO_2_ samples were measured with a Jasco V550 UV/Vis spectrophotometer (Jasco, Inc., Tokyo, Japan).

Pure and N-doped TiO_2_ nanoparticles were autoclaved and dispersed in DMEM-H medium at a concentration of 100 μg/ml, respectively. The samples were ultrasonicated for 15 min before using.

### Cell culture and PDT treatment

The human cervical carcinoma cells (HeLa) procured from the Cell Bank of Shanghai Science Academy were grown in Petri dishes in DMEM-H solution supplemented with 10% fetal calf serum in a fully humidified incubator at 37°C with 5% CO_2_ for 24 h.

The cells were incubated with 100 μg/ml pure or N-doped TiO_2_ under light-free conditions for 2 h and were then illuminated with a visible light filtered by a bandpass filter (400 to 440 nm) from a Xe lamp (100-W; Olympus, Center Valley, PA, USA) at a power density of 40 mW/cm^2^ for 5 min. The transmission spectrum of that bandpass filter was shown in Additional file 2: Figure S2. As shown in the figure, the filter could transmit some light with the wavelength below 400 nm. Therefore, the pure TiO_2_ could still absorb a small amount of the transmitted light.

### Measurement of ROS induced by TiO_2_ or N-TiO_2_ in aqueous suspensions

For the measurement of photo-induced ROS in TiO_2_ or N-TiO_2_ aqueous suspensions, 2^′^,7^′^-dichlorfluorescein (DCFH), was used as a probe. The DCFH was converted from the diacetate form DCFH (DCFH-DA) (Sigma-Aldrich) by adding 0.5 ml of 1 mM DCFH-DA in methanol into 2 ml of 0.01 N NaOH and keeping the mixture at room temperature in the dark for 30 min. It was then neutralized with 10 ml sodium phosphate buffer (pH = 7.2) [21]. Pure or N-doped TiO_2_ in phosphate buffered saline (PBS, 100 μg/ml) were mixed with DCFH (25 μM) before visible light irradiation. The non-fluorescent DCFH can rapidly react with ROS to form fluorescent 2^′^,7^′^-dichlorofluorescein (DCF). By measuring the fluorescent intensity, the production of ROS could be estimated.

To measure the generations of specific ROS, two probes were used respectively. Dihydrorhodamine 123 (DHR) is mainly sensitive to O_2_^ ·−^[22] and H_2_O_2_[23], and 2-[6-(4-aminophenoxy)-3-oxo-3H-xanthen-9-yl]-benzoic acid (APF) is selectively sensitive to OH · [23]. It was already demonstrated that the reactive species H_2_O_2_, O_2_^ ·−^, and ^1^O_2_ did not cause any modification in the fluorescence of the probe APF [24]. Pure or N-doped TiO_2_ in PBS (100 μg/ml) were mixed with DHR (25 μM, Sigma-Aldrich) or APF (10 μM, Cayman Chemical, Ann Arbor, MI, USA) before irradiation. Upon oxidation, the non-fluorescent DHR or APF is converted to the highly fluorescent Rhodamine 123 or fluorescein.

After the samples were irradiated by a visible light (400 to 440 nm) with a power density of 40 mW/cm^2^ for different times ranging from 1 to 5 min, the fluorescence spectra were recorded by a spectrometer (F-2500, Hitachi, Brisbane, CA, USA) and the fluorescent intensities were compared.

### MMP assay

Rhodamine 123 [2-(6-amino-3-imino-3H-xanthen-9-yl) benzoic acid methyl ester] (Beyotime, Jiangsu, China), which could bind specifically to the mitochondria, was used to estimate the MMP. When MMP is decreased, the dye could be released from the mitochondria and the fluorescence vanished. The PDT-treated cells were incubated with Rhodamine 123 (5 μg/ml) for 30 min in the dark at 37°C and then were washed with Dulbecco’s PBS (D-PBS) for three times before the visible light illumination.

### Measurement of Ca^2+^ concentration

To study the intracellular calcium concentration, HeLa cells were loaded with 10 μM Fluo-3 AM (Beyotime) for 30 min at 37°C and followed by washing with D-PBS for three times. Then the cells were incubated for another 20 min to ensure complete cleavage of Fluo-3 AM by the intracellular ester enzyme that releases Fluo-3 before the illumination.

### Measurement of intracellular NO

The intracellular NO level was detected using a NO-sensitive fluorescence probe DAF-FM DA [3-amino, 4-aminomethyl-2^′^,7^′^-difluorescein, diacetate] (Beyotime). The cells were loaded with 10 μM DAF-FM DA at 37°C in the kit buffer for 20 min and were then gently washed with D-PBS for three times and incubated for another 20 min to ensure that the intracellular DAF-FM DA was completely catalyzed to form DAF-FM by ester enzyme before the illumination.

### Cell morphology and cytoskeleton observation

The HeLa cells were fixed with 4% paraformaldehyde for 15 min at room temperature with different time intervals after the illumination. Then they were permeabilized with 0.025% Triton X-100 in D-PBS (Sigma-Aldrich Corp., St. Louis, MO, USA) for 2 min. After washing with D-PBS three times, the cells were treated with 1% bovine serum albumin (BSA) for 2 h at 4°C. The fixed cells were stained with 5 nM Alexa Fluor® 488 phalloidin conjugate (Invitrogen, Eugene, OR, USA) for F-actin labeling for 40 min at 37°C. Meanwhile, 1% BSA was added to the staining solution to reduce nonspecific background staining. The cells were washed with 0.05% PBS-Tween20 three times before microscopic observation.

### Microscopy and image analysis

The fluorescence images of cells were observed by a laser scanning confocal microscope (FV-300, IX71; Olympus, Tokyo, Japan) using a 488-nm continuous wave Ar^+^ laser (Melles Griot, Carlsbad, CA, USA) as the excitation source and a × 60 water objective to focus the laser beam. A 505- to 550-nm bandpass filter was used for the fluorescence images. Each experiment was repeated three times independently.

The fluorescence intensities of MMP, Ca^2+^, and NO probes from the microscopic images were analyzed with the Olympus Fluoview software. The data were expressed in terms of the relative fluorescence intensity of the probes and expressed as mean ± SD. The fluorescence intensity was averaged from 100 to 150 cells for each experiment.

## Results and discussion

### Generation of ROS by pure and N-doped TiO_2_ in aqueous suspensions

**Figure 1:**
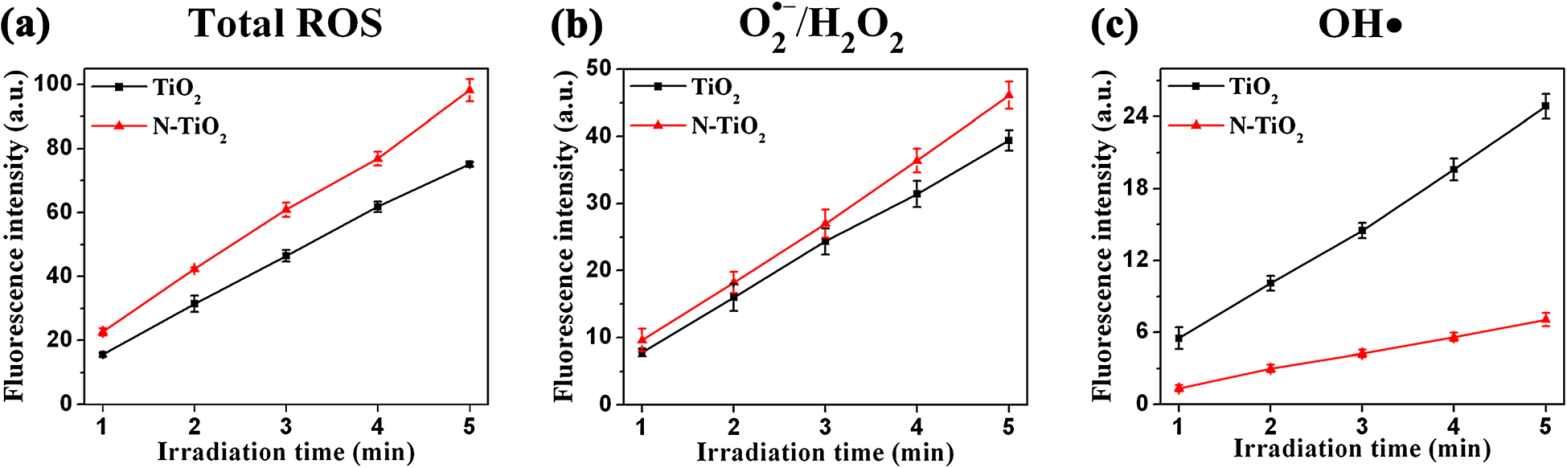
**Comparison of ROS induced by TiO_**2 **_and N-TiO_**2**_. **Fluorescence measurements as a function of irradiation time to compare the productions of ROS and specific ROS in aqueous suspensions induced by TiO_2_ and N-TiO_2_: (**a**) total ROS, (**b**) O_2_^·−^/H_2_O_2_, and (**c**) OH · .

The generations of ROS induced by TiO_2_ or N-TiO_2_ nanoparticles in aqueous suspensions under visible light irradiation were studied using the fluorescence probes as described in the ‘Methods’ section. The fluorescence intensities with the irradiation times ranging from 1 to 5 min were shown in Figure 1a. The fluorescence intensities of both TiO_2_ (the black line) and N-TiO_2_ (the red line) samples increased with irradiation time but the fluorescence intensities of N-TiO_2_ samples were always higher than that of the TiO_2_ ones. It means that N-TiO_2_ could generate more ROS than TiO_2_ under visible light irradiation, which agrees well with the spectral result that N-TiO_2_ showed higher visible light absorption than TiO_2_ (see Additional file 1: Figure S1, where a shoulder was observed at the edge of the absorption spectra, which extended the absorption of N-TiO_2_ from 380 to 550 nm).

The major reactions for the formation of ROS upon illumination of TiO_2_ have been proposed as follows [25]:

(1) $${\mathrm{TiO}}_{2}+\mathrm{hv}\left(\geq E_{g}\right)\text{→}h^{+}+e^{-}$$

(2) $$H_{2}O+h^{+}\text{→}\mathrm{OH}\cdot +H^{+}$$

(3) $$2\mathrm{OH}\cdot \text{→}H_{2}O_{2}$$

(4) $$O_{2}+e^{-}\text{→}O_{2}{}^{\cdot -}$$

(5) $$O_{2}{}^{\cdot -}+h^{+}{\text{→}}^{1}O_{2}$$

(6) $$H_{2}O_{2}+O_{2}{}^{\cdot -}\text{→}\mathrm{OH}\cdot +{\mathrm{OH}}^{-}+O_{2}$$

OH · is mainly formed in the reaction of photogenerated holes with surrounding water, while O_2_^ ·−^ is formed in the reaction of photogenerated electrons with dissolved oxygen molecules. Some O_2_^ ·−^ can form ^1^O_2_ by reacting with the holes. Moreover, some OH · can form H_2_O_2_, and the reactions of H_2_O_2_ can also result in the formation of OH · with a lesser extent.

Since DCFH is a nonspecific ROS probe, it is necessary to further analyze the specific ROS. As described above, DHR and APF were used to evaluate the generation of O_2_^ ·−^/H_2_O_2_ and OH·, respectively. The fluorescence measurements in Figure 1b,c shows that all the specific ROS increased with the irradiation time, but the N-TiO_2_ induced more O_2_^ ·−^/H_2_O_2_ (Figure 1b) while less OH · (Figure 1c) than TiO_2_. It was reported that the photogenerated holes of N-TiO_2_ were trapped in the N 2*p* levels and had a very low mobility [26], thus were barely involved in the photocatalysis when the N-TiO_2_ was illuminated by visible light [27]. In this study, the lower production of OH · from N-TiO_2_ might result from the same reason. However, the photogenerated electrons in the conduction band can react with oxygen molecules to generate O_2_^ ·−^, which is thermodynamically favored [28]. Thus, N-TiO_2_ could generate more O_2_^ ·−^/H_2_O_2_ than the pure TiO_2_ due to the higher visible light absorption efficiency.

When cells were treated with TiO_2_ or N-TiO_2_ nanoparticles, the nanoparticles were not only found on the cell membrane but also in the cytoplasm, and some of them aggregated around or in Golgi complexes and even in nuclei [10]. As the TiO_2_ or N-TiO_2_ nanoparticles can induce ROS under visible light irradiation, the photokilling effect on cancer cells was observed in our previous work [10]. Considering that the productions of the specific ROS species generated by TiO_2_ or N-TiO_2_ are different and the contributions from the specific ROS to PDT may also be different, the PDT-induced changes of the intracellular parameters, such as MMP, Ca^2+^, and NO concentrations in HeLa cells treated with TiO_2_ or N-TiO_2_ were studied as follows.

### MMP changes

**Figure 2:**
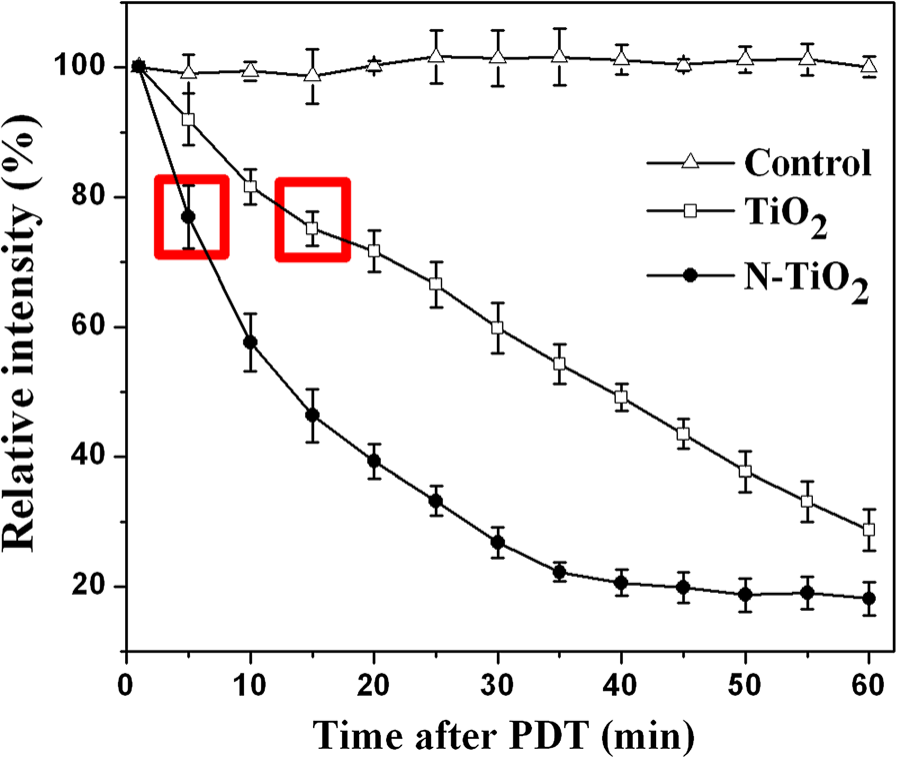
**MMP of HeLa cells as a function of the time after the PDT. **Cells were incubated with 100 μg/ml TiO_2_ (white square) or N-TiO_2_ (black circle) for 2 h and illuminated by the visible light for 5 min. The averaged fluorescence intensity of control cells (white triangle) at 0 min was set as 100%.

When TiO_2_- or N-TiO_2_-treated cells were illuminated by light, the generated ROS may attack the mitochondria [29] or the activated nanoparticles may interact with the mitochondria directly [30], which would affect the function of mitochondria and cause the opening of mitochondrial permeability pores, resulting in the dissipation of MMP [30-32]. In this study, the MMP decreased immediately after the PDT as shown in Figure 2. It seems that the mitochondrion is a very sensitive cellular organelle during the PDT, and the defects can be detected immediately in our study. For TiO_2_-treated cells, the MMP level decreased continuously after the PDT with an approximate rate of 1.2% per min within 60 min. The MMP level for N-TiO_2_ samples dropped much faster (around 4.2% per min) within the first 10 min after the PDT, then decreased at slower and slower rate within 45 min, and almost kept in a constant rate of 20% after 45 min. However, the MMP levels of control cells and the cells incubated with TiO_2_ and N-TiO_2_ under light-free conditions did not show any change during 60 min (data not shown), which confirmed the low cytotoxicity of TiO_2_ and N-TiO_2_.

It should be noted that the MMP level of N-TiO_2_-treated cells decreased 3.5 times faster than that of the TiO_2_-treated cells at the beginning after the PDT. Compared with Figure 1c that there were considerably more OH · induced by TiO_2_ than N-TiO_2_ under visible light, it strongly suggested that the hydroxyl radicals with the rather shorter lifetime and lower diffusion length than O_2_^ ·−^ and H_2_O_2_[33] might contribute less on the damage of mitochondria among a variety of ROS in PDT.

### Intracellular Ca^2+^ concentration

It has been reported that some signal transduction pathways were activated by PDT [34]. Calcium expression level was one of the concerning principal factor since it is an important link between the pathways. The activation of Ca^2+^ was also known as a contributor to the cell morphological and functional changes associated with apoptosis [35]. The raise of intracellular calcium levels would result in various changes of cellular metabolism as well as the cell morphology.

**Figure 3:**
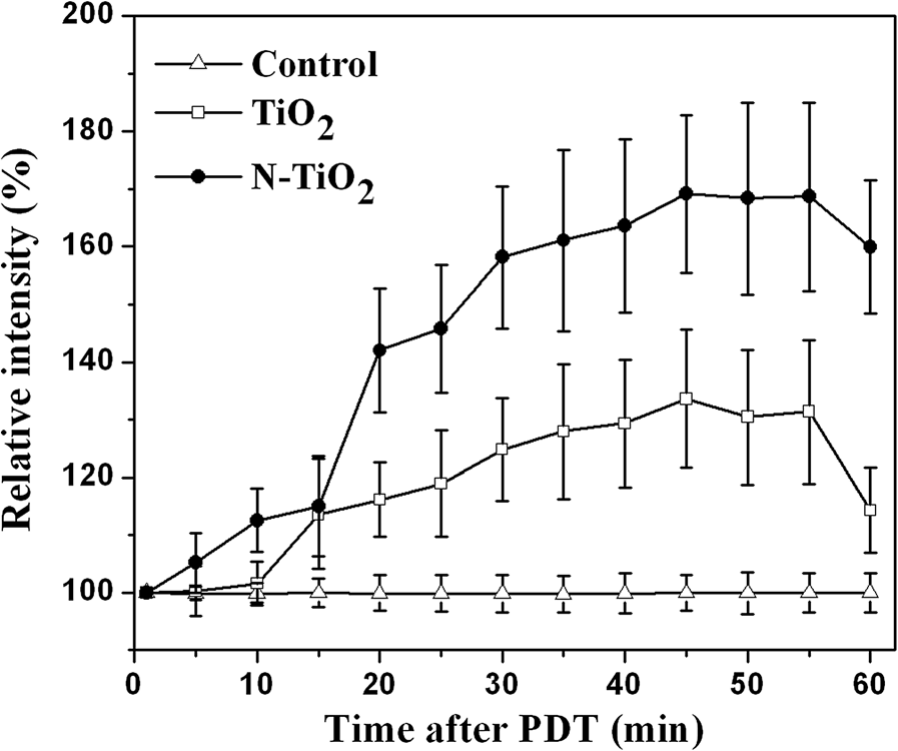
**Time**-**dependent changes of the intracellular Ca**^**2+ **^**levels after the PDT. **The averaged fluorescence intensity of control cells (white triangle) was set as 100%. TiO_2_ (white square)- or N-TiO_2_ (black circle)-treated cells (100 μg/ml) were incubated under light-free conditions for 2 h and illuminated by the visible light for 5 min.

The time-dependent intracellular Ca^2+^ concentrations after the PDT were measured as shown in Figure 3. The detectable increase of the intracellular Ca^2+^ levels for TiO_2_ samples was first observed at 15 min after the PDT, while that for N-TiO_2_ samples, it was observed at the first measurement point of 5 min after the PDT. Comparing the data in Figure 3 with that in Figure 2, we can see the elevation of Ca^2+^ followed by the loss of MMP. To demonstrate the correlativity of Ca^2+^ and MMP, the starting times of the detectable increase of Ca^2+^ were marked as two red squares in Figure 2. It suggests that a certain amount of the MMP loss (about 24% ± 5%) would cause the detectable increase of Ca^2+^.

As shown in Figure 3, the Ca^2+^ levels for both TiO_2_ and N-TiO_2_ samples reached the maximum values at about 45 min after the PDT, where N-TiO_2_ induced release of Ca^2+^ at around 2.1-fold than TiO_2_ did. Since there was no calcium ion in the D-PBS solution, the detected Ca^2+^ might be released from the damaged calcium stores, such as mitochondria and possibly other organelles, and flow into the cytoplasm through ion channels [36].

This result agreed with the data of MMP changes. The MMP levels of N-TiO_2_ decreased around 3.5 times faster than that of TiO_2_ at the early time after the PDT, which means the N-TiO_2_ induced damage of mitochondria was more serious. Therefore, the released Ca^2+^ could be observed earlier and the Ca^2+^ levels were higher in N-TiO_2_ samples as compared to the TiO_2_ samples.

### Generation of NO

**Figure 4:**
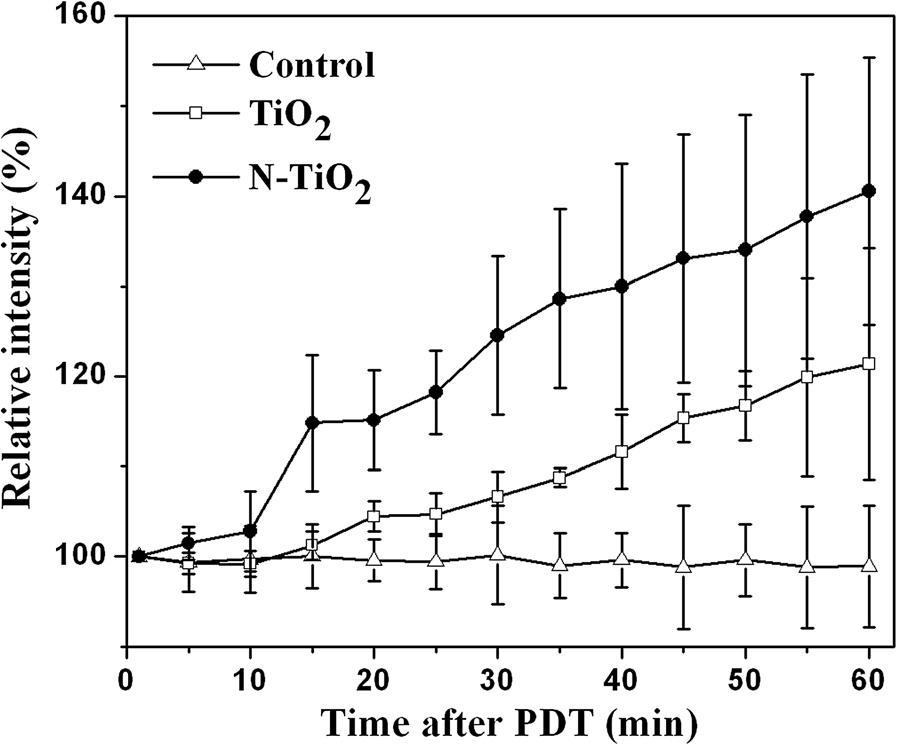
**Changes of the intracellular NO levels as a function of the time after the PDT. **The averaged fluorescence intensity of control cells (white triangle) was set as 100%. TiO_2_ (white square)- or N-TiO_2_ (black circle)-treated cells were incubated with 100 μg/ml under light-free conditions for 2 h before the irradiation.

The cells have defense mechanisms such as the endogenous generation of NO, which can scavenge a certain amount of ROS and protect cells from ROS attack [32,36,37]. The change of the NO level after the PDT was also detected in this work. The intracellular NO levels of N-TiO_2_ samples increased faster than that of the TiO_2_ ones (Figure 4), the former increased from 100% (as control cells) to 141% in 60 min after the PDT, while the latter increased to 121% only. It means that more NO was generated to buffer the increased ROS under higher oxidative stress for N-TiO_2_ samples although TiO_2_ induced higher amount of OH·. This result also suggested that the OH· species played a less important role among a variety of ROS in the PDT. Taken the above findings together, it suggested that the ROS overwhelmed the antioxidant defense capacity of NO in the cells, although NO could buffer the ROS to a certain extent. The remaining ROS would become highly harmful and lead to irreversible cellular damage.

### Cell morphology and cytoskeleton defects

**Figure 5:**
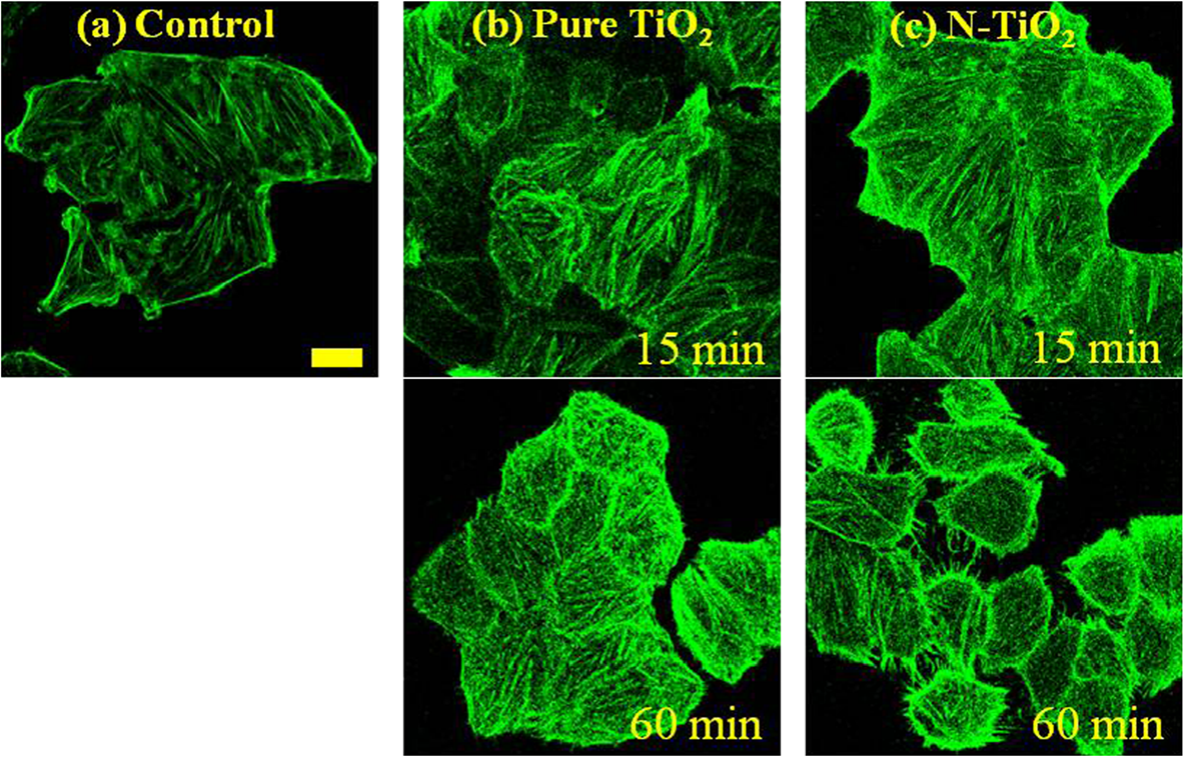
**The morphology and cytoskeleton of HeLa cells at different time points after the PDT. **(**a**) Control cells. (**b**) TiO_2_-treated cells. (**c**) N-TiO_2_-treated cells (scale bar, 20 μm). Cells were incubated with 100-μg/ml TiO_2_ or N-TiO_2_ under light-free conditions for 2 h before the PDT and then fixed at 15 min and 60 min after the PDT, respectively. The cells were stained with Alexa Fluor® 488 phalloidin for F-actin.

The cell morphology images of HeLa cells at different times after the PDT were acquired by a confocal microscope with the labeled F-actin. No morphology and cytoskeleton defects were found at 15 min after the PDT for both TiO_2_ and N-TiO_2_ samples (Figure 5b,c, upper images)_._ At 60 min after the PDT, the organization of actin cytoskeleton of the cells incubated with TiO_2_ seemed disrupted (Figure 5b, lower image), while the cells incubated with N-TiO_2_ exhibited serious distortion and membrane breakage (Figure 5c, lower image).

As ROS can be generated around TiO_2_ or N-TiO_2_, the nanoparticles near the cell membranes may directly cause cell membrane damage by biochemical reactions. Additionally, the PDT-induced defect of mitochondria and the release of Ca^2+^ into the cytoplasm might trigger cell apoptosis or necrosis, which may result in the cell morphology and cytoskeleton defects eventually. As the cytoskeleton is involved in many intracellular signaling pathways, the cytoskeletal distortion and shrinkage need to be further studied for a long observation time in future studies.

## Conclusions

A comparison of the killing effects between N-TiO_2_ and TiO_2_ on HeLa cells with visible light irradiation was conducted. N-TiO_2_ produced more ROS and specifically more O_2_^ ·−^/H_2_O_2_ under visible light irradiation. Contrarily, more OH · were produced by TiO_2_. The MMP levels were sensitive in the PDT, and rapid loss of MMP was detected at the very beginning after the PDT as one of the earliest detectable biochemical changes in this study. A certain amount of MMP loss (around 24%) was followed by the detectable increase of Ca^2+^ (at 5 and 15 min after the PDT for N-TiO_2_ and TiO_2_, respectively). The increase of NO was detected later than the other intracellular parameters, which indicates that the NO generation was caused by the generation of ROS. The N-TiO_2_ resulted in more loss of MMP and higher increase of Ca^2+^ and NO in HeLa cells and, finally, induced more cell damages than pure TiO_2_. At 60 min after irradiation, significant cytoskeletal shrinkage and breakage were observed for N-TiO_2_-treated cells, whereas for TiO_2_-treated cells, only slight damage was demonstrated. Overall, N-TiO_2_ can induce more cell damages than pure TiO_2_. The hydroxyl radicals might contribute less to the cell damages among a variety of ROS.

## Competing interests

The authors declare that they have no competing interests.

## Authors’ contributions

ZL carried out the experiments and drafted the manuscript. XP and TW participated in the confocal microscopy imaging. PW supervised the work, participated in the discussion of the results and in revising the manuscript. JC participated in the discussion of the results. LM designed the project and wrote the manuscript. All authors read and approved the final manuscript.

## Supplementary Material

Additional file 1: Figure S1 Absorbance spectra of TiO_2_ and N-TiO_2_ nanoparticles. Description: A shoulder was observed at the edge of the absorption spectra, which extended the absorption of N-TiO_2_ from 380 nm to 550 nm. Click here for file

Additional file 2: Figure S2 The transmission spectrum of the 400 to 440 nm bandpass filter. Description: The filter could transmit some light with the wavelength below 400 nm, which could be absorbed by the pure TiO_2_ as shown in Additional file 1: Figure S1. Click here for file
